# HDMS-YOLO: a multi-scale weed detection model for complex farmland environments

**DOI:** 10.3389/fpls.2025.1696392

**Published:** 2025-10-22

**Authors:** Jing Hua, Ruimin He, Yanhua Zeng, Qi Chen

**Affiliations:** ^1^ School of Software, Jiangxi Agricultural University, Nanchang, China; ^2^ School of Agriculture, Jiangxi Agricultural University, Nanchang, China; ^3^ School of Computer Science, Jiangxi Agricultural University, Nanchang, China

**Keywords:** weed detection, deep learning, multi-scale feature fusion, YOLO, precision agriculture

## Abstract

**Introduction:**

With the continuous advancement of agricultural technology, automatic weed removal has become increasingly important for precision agriculture. However, accurate weed identification remains challenging due to the diversity and varying sizes of weeds, as well as the high visual similarity between weeds and crops in terms of shape, colour, and texture.

**Methods:**

To address these challenges, this study proposes the HDMS-YOLO model for robust weed identification, trained and evaluated on the publicly available CropAndWeed dataset. The model incorporates two novel feature extraction modules—the Shallow and Deep Receptive Field Distillation (SRFD and DRFD) modules—to effectively capture both shallow and deep weed features. The traditional C3K2 structure is replaced by the Partial Convolution-based Multi-Scale Feature Aggregation (PC-MSFA) module, which enhances feature representation through partial convolution and residual connections. In addition, a new IntegraDet dynamic task-alignment detection head is designed to further improve localisation and classification accuracy.

**Results:**

Experimental results show that HDMS-YOLO achieves an accuracy of 74.2%, a recall of 66.3%, and an mAP of 71.2%, which are 2.6%, 2.1%, and 2.6% higher, respectively, than those of YOLO11. Compared with other mainstream algorithms, HDMS-YOLO demonstrates superior overall detection performance.

**Discussion:**

The proposed HDMS-YOLO model exhibits strong capability in extracting and representing weed features, leading to improved identification accuracy and generalisation. These results highlight its potential application in precision farm management and the development of intelligent weed-removal robots for unmanned agricultural systems.

## 1 Introduction

Weeds are invasive plants that compete with crops for essential resources like water, nutrients, sunlight, and space, which results in reduced crop yields and hindered growth. Additionally, weeds often act as hosts for pests and diseases, further exacerbating crop losses (Zhu et al., 2020; Vasileiou et al., 2024; Wang et al., 2025). As a result, early-stage weed control is vital to preserve agricultural productivity and minimize crop yield losses (Reuter et al., 2025). Traditional methods, such as manual labor and chemical herbicide application, are resource-intensive, costly, and environmentally damaging due to pesticide residues and pollution risks (Yang et al., 2024; Munir et al., 2024; Yu et al., 2025). Precision spraying robots can achieve large-scale pesticide application on weeds, effectively preventing issues related to chemical waste and pesticide residue. Accurate weed detection is crucial for achieving precise pesticide application to weeds (Upadhyay et al., 2024; Zhang et al., 2023). With the widespread application of artificial intelligence technology in agriculture, researchers have increasingly applied deep learning methods to weed identification and detection. Mesías et al. combined drone images with the convolutional neural network model Inception-ResNet-v2 to advance methods for identifying weeds in their early growth stages, further promoting the precise and efficient implementation of SSWM technology (Mesías-Ruiz et al., 2024). Veeragandham et al. used the AlexNet, VGG-16, VGG-19, ResNet-50, and ResNet-101 models to classify and compare 15 common weed species in a peanut crop dataset, finding that the accuracy rate on VGG-19 without frozen layers exceeded 99% (Veeragandham and Santhi, 2022). Duong et al. were able to achieve automatic and highly accurate detection of weeds through EfficientNet and transfer learning (Duong et al., 2024). Jian et al. proposed a method for identifying weeds during the seedling stage of soybeans using drone data and deep learning algorithms (Cui et al., 2024).

Two-stage object detection method. Although they achieve high accuracy, two-stage models often fail to meet the real-time requirements for weed detection and localization. Two-stage object detection requires generating candidate boxes in advance before performing classification and regression. This process is particularly computationally intensive when dealing with high-resolution images or scenarios that generate numerous candidate boxes, resulting in a significant decrease in the model’s inference speed and making it unsuitable for real-time weed detection applications with high-performance requirements. In contrast, single-stage object detectors do not require pre-generated candidate boxes and can directly predict weed categories and bounding boxes on feature maps. The model structure of single-stage object detectors is simpler than that of two-stage models, making them easier to run on edge devices and suitable for real-time weed detection applications. Zheng et al. improved YOLOv8 by utilizing Star Blocks and LSCSBD heads, which reduced the parameters by 50% and the model size by 47%, achieving a model detection accuracy of 98% mAP@50 and 95.4% mAP@50–95 on the CottonWeedDet12 dataset (Lu et al., 2025). Li et al. enhanced the YOLOv8 and DINO models using mainstream improvement strategies. They validated the model’s effectiveness by constructing a new winter wheat weed (3W) dataset (Li et al., 2024). Fan et al. proposed the YOLO-WDNet model, which replaces CSPDarknet53 with ShuffleNet v2 to reduce the model’s FLOPs. The PHAM mechanism and the improved BiFPN in the model facilitate the extraction of plant features in complex scenes, thereby enhancing the model’s accuracy (Fan et al., 2024). Feng et al. proposed a 12-class cotton image dataset and evaluated the impact of data augmentation on weed detection by comparing 18 YOLO models (Dang et al., 2023). Chen et al. proposed a YOLO-based sesame weed detection model, YOLO-Sesame, by incorporating an attention mechanism into the SPP structure, utilizing the SE block to enhance local important pooling, and integrating the ASFF structure to address false negatives, thereby effectively improving detection accuracy (Chen et al., 2022). Ma et al. proposed YOLO-CWD, which employs a novel hybrid attention mechanism and loss function to enhance model recognition accuracy, resulting in significant improvements in detection performance on corn and weed datasets (Ma et al., 2025). Goyal et al. validated the YOLOv8 and Mask RCNN models on potato plant and weed datasets, demonstrating that the model can detect weeds in highly complex and severely occluded environments (Goyal et al., 2025). Xu et al. integrated YOLOv5 with the Vision Transformer to propose the W-YOLOv5 crop detection algorithm (Xu et al., 2024).

The presence of various-sized weed targets has limited the accuracy of the model. Especially for crops and weeds with similar shapes, it cannot effectively distinguish them in complex environments. To address this issue, this study proposes the HDMS-YOLO model. It evaluates it on the CropAndWeed dataset. Firstly, in the feature extraction part, the structured reconstruction module of shallow features (SRFD) and the dynamic reconstruction module of deep features (DRFD) are introduced to form a hierarchical feature processing mechanism, thereby effectively enhancing the perception ability of the model for targets at different scales. The PC-MSFA module enhances weed detection across varying scales via cross-stage partial connections and progressive multi-scale feature aggregation. Thirdly, IntegraDet enhances the model’s ability to distinguish morphologically similar crops from weeds by dynamically adjusting the loss weights for classification and regression tasks. The proposed HDMS-YOLO detection model can accurately and in real-time identify the types of weeds.

## 2 Materials and methods

### 2.1 Crop and weed dataset

**Figure 1:**
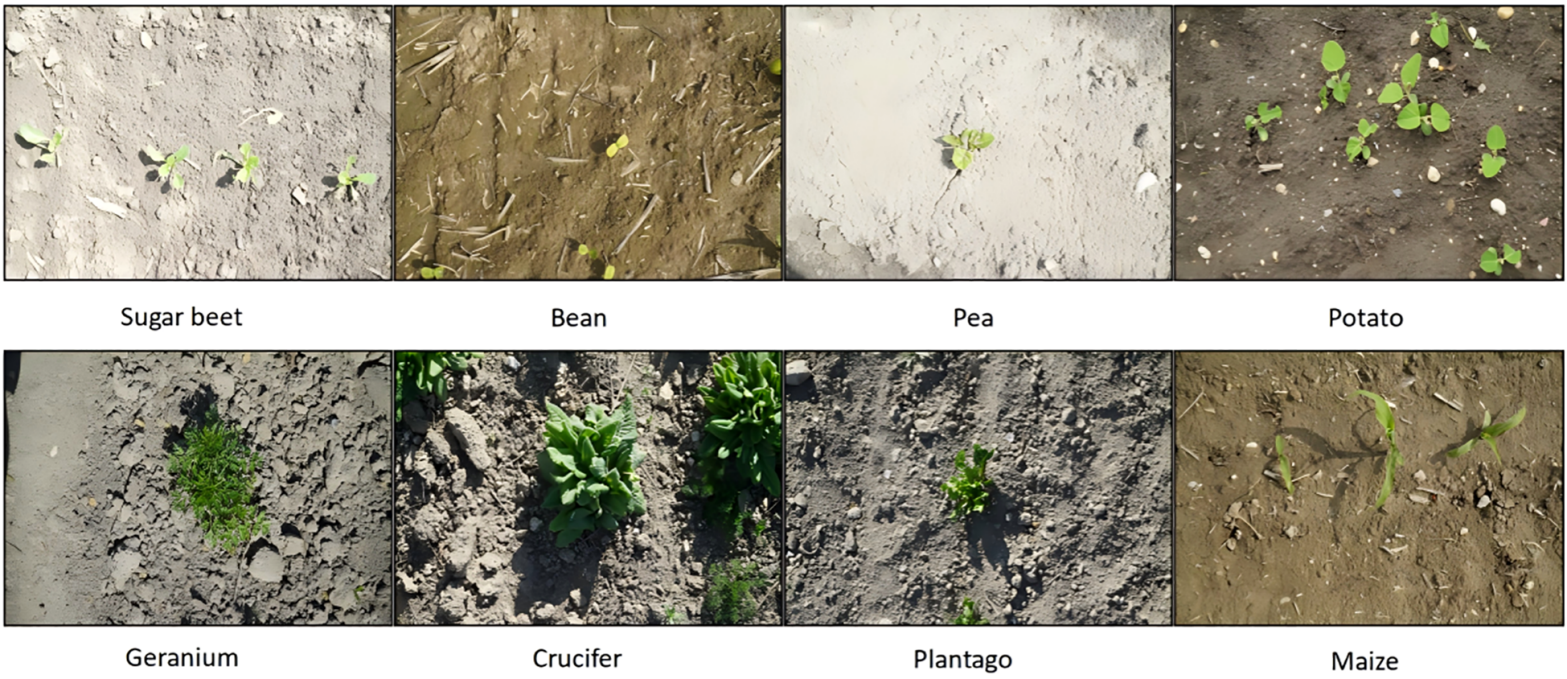
Display of some dataset data.

**Figure 2:**
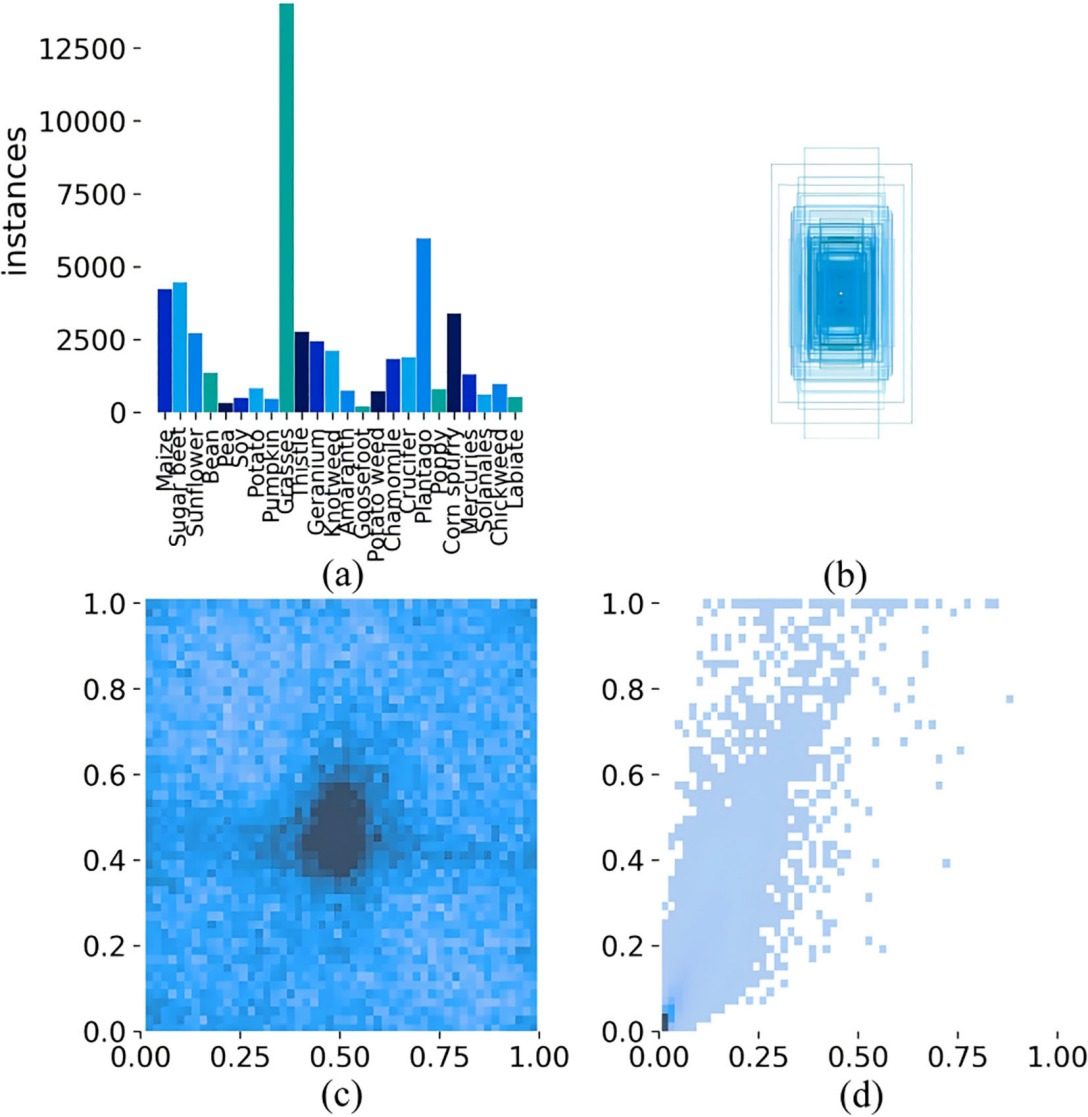
**(a)** Statistics and separation of data annotation files (Data annotation weed - species histogram); **(b)** Distribution map of the length and width of the dataset annotation box; **(c)** Histogram of the dataset variables x and y; **(d)** Histogram of the width and height of the dataset variables.

This study used the CropAndWeed dataset to train and evaluate the model (Steininger et al., 2023). Researchers collected and annotated the dataset from hundreds of commercial croplands in Austria. They photographed many unique species in a controlled outdoor environment and divided the dataset into multiple variants, each containing different object classes. This study used the ‘Fine24’ variant of the dataset. This variant comprises 24 plant classes, consisting of eight crop species and 16 weed species. This dataset consists of 7,705 images, which were divided into three parts in the ratio of 7:1:2: the training set, the validation set, and the test set, containing 5,393, 770, and 1,542 images, respectively. The dataset includes images captured under different lighting conditions, soil types, and humidity levels. To provide a comprehensive overview of the distribution of each category in the dataset, **Figure 1** shows some examples. **Figure 2** displays the quantity and distribution characteristics of various weed species.

### 2.2 Experimental setup

This experiment was conducted on a remote server. The detailed computer software, hardware configuration and training environment settings are shown in **Table 1**. This model was trained using the CropAndWeed dataset. We set the batch size to 16, the number of epochs to 300, the initial weights to random weights, seed to 0, Momentum to 0.937, optimizer to SGD, learning rate to 0.01, workers to 8, and seed to 0. The size of the input images was adjusted to 640 x 640 pixels.

**Table 1:** Experimental conditions.

Device	Version
GPU	NVIDIA GeForce RTX 2080 Ti
Memory	11GB
Frame	Pytorch 2.2.2
Tool	Python 3.10.14
GPU accelerator	CUDA 12.2

### 2.3 Construction of weed detection model based on MMDetection

MMDetection is an open-source object detection toolbox jointly developed by the Multimedia Laboratory of the Chinese University of Hong Kong (CUHK-MMLab) and SenseTime. It encapsulates dataset construction, model building, and training strategies into individual modules. We can implement a new algorithm with a small amount of code through module invocation, significantly enhancing code reusability. A notable advantage of MMDetection is its fast training speed. In recent years, it has been widely used in commercial research for detecting moving and stationary objects, achieving higher accuracy than other object detection frameworks. Built with PyTorch and CUDA, MMDetection provides powerful, fast, and highly accurate results. Its products include well-known models such as RetinaNet50, Fast R-CNN, and Faster R-CNN.

In our study, we employed RetinaNet along with Faster R-CNN. The model employs FPN as its head, with ResNet-50 and ResNet-101 serving as the backbone (Chen et al., 2019). **Table 2** provides a summary of the training parameters. These parameters include iteration (the number of training iterations), Batch size (the number of data samples per iteration), learning rate (LR), and Maximum size (the maximum input image size).

**Table 2:** Model structures and some hyperparameter settings based on MMDetection.

Model	Backbone	Head	Num workers	Iterations(steps)	Batch size	Learning rate	Max size (px)
RetinaNet	Resnet50	FPN	8	10000	8	0.01	640
		FPN	8	60000	8	0.01	640
	Resnet101	FPN	8	10000	8	0.01	640
		FPN	8	60000	8	0.01	640
Faster R-CNN	Resnet50	FPN	8	10000	8	0.01	640
		FPN	8	60000	8	0.01	640
	Resnet101	FPN	8	10000	8	0.01	640
		FPN	8	60000	8	0.01	640

### 2.4 Construction of a weed detection model based on YOLO11

This study utilizes the YOLO11n model. YOLO11 is a target detection model in the YOLO family, supporting detection, classification, and segmentation tasks (Khanam and Hussain, 2024). The neck module of YOLO11 is an improvement based on the concepts of Feature Pyramid Network (FPN) and Path Aggregation Network (PAN). YOLO11 replaces the C2f module in the neck with the C3k2 module, aiming to achieve faster speed and higher efficiency, thereby enhancing the overall performance of the feature aggregation process. Additionally, the C2PSA module in the model enables it to focus on key areas in the image, significantly improving its understanding of complex scenes. YOLO11 utilizes a decoupled detection head to output prediction results from three feature maps at varying scales, corresponding to different granular levels of the image. This approach detects small targets at a finer level while capturing large targets through higher-level features.

**Figure 3:**
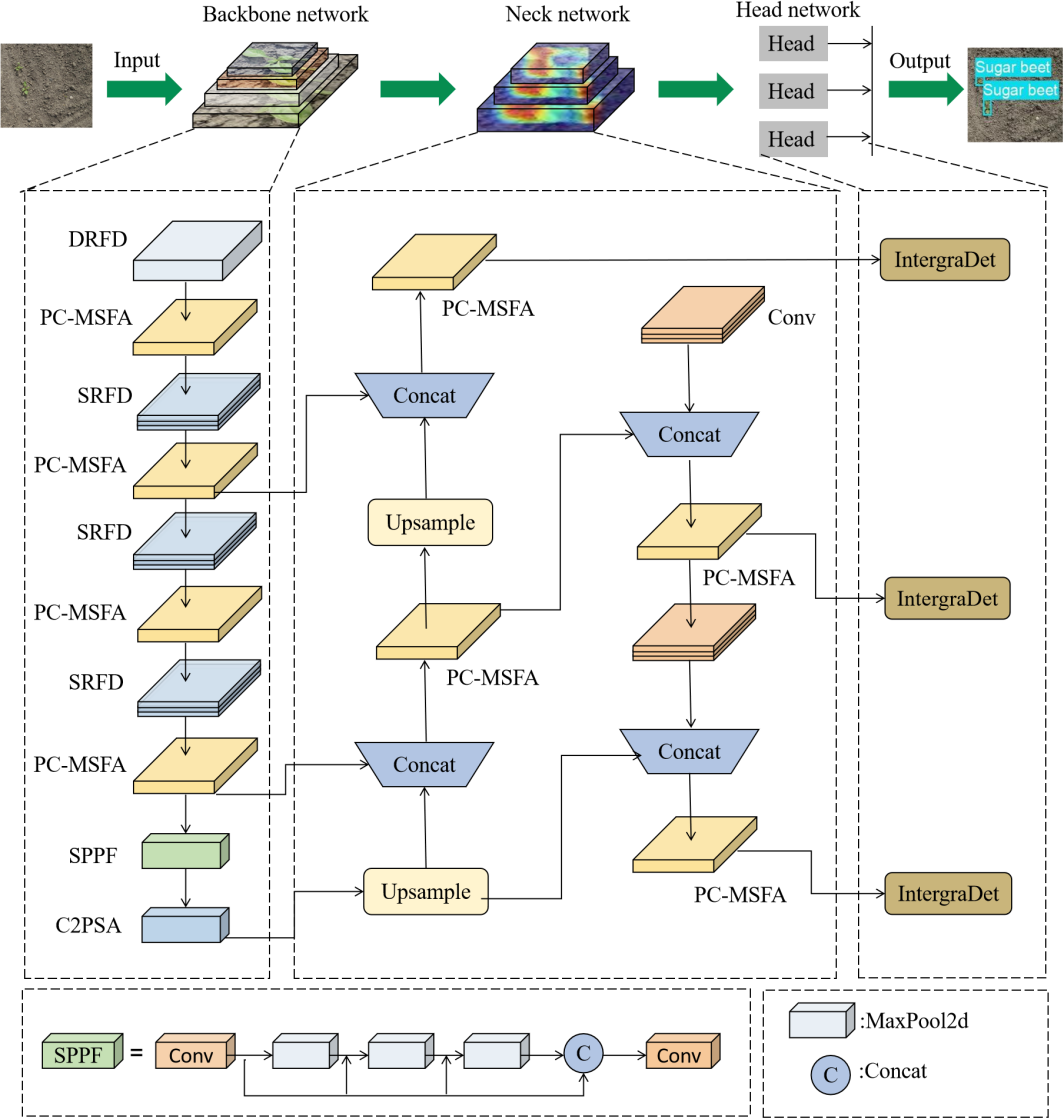
The overall architecture of the HDMS-YOLO network.

Although the small size of YOLO11 guarantees speed and efficiency, its accuracy is relatively limited. To address this issue, we developed an improved HDMS-YOLO model that can accurately detect various weeds and crops under complex weather conditions. Firstly, shallow feature extraction SRFD and deep feature extraction DRFD modules (Lu et al., 2023) are introduced in the feature extraction part to replace the convolutional modules in the backbone network, thereby improving the model’s capability to extract image features during detection processes. Secondly, we propose a PC-MSFA module that combines partial convolution and residual connections to expand the receptive field and enhance the expressive power of the model. Finally, we propose a dynamic task alignment integrated detection head to enhance target capture capability, significantly improving detection performance. HDMS-YOLO achieves higher detection accuracy in crop and weed detection tasks. **Figure 3** shows its architecture. The following section provides detailed explanations.

#### 2.4.1 HRFN module

**Figure 4:**
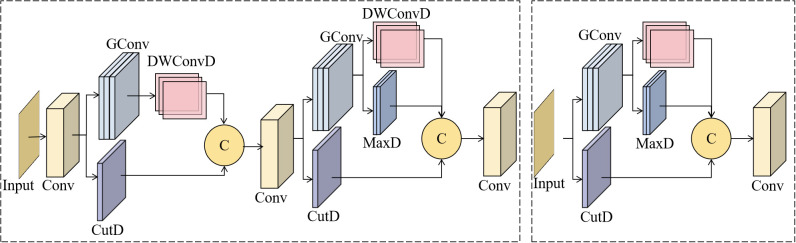
SRFD and DRFD module diagrams.

Traditional convolutional downsampling in object detection networks often leads to the loss of crucial spatial details, which is particularly problematic for detecting small weeds in agricultural fields. To address this issue, we propose the Hierarchical Robust Feature Network (HRFN), as shown in **Figure 4**, which replaces conventional downsampling layers in the YOLO11 backbone with two specialized modules: Shallow Robust Feature Downsampling (SRFD) and Deep Robust Feature Downsampling (DRFD).

The SRFD module processes input images through a two-stage downsampling pipeline. Initially, a 7×7 convolution extracts preliminary features while preserving spatial resolution. In the first stage, the image size is halved using two parallel pathways: CutD, which retains spatial features through slicing, and ConvD, which employs grouped convolutions to extract local features. These features are then fused to retain both structural and semantic information. In the second stage, the resolution is reduced to a quarter, employing three parallel branches: ConvD for context, MaxD for prominent features, and CutD for spatial details, ensuring comprehensive feature preservation during the downsampling process.

The DRFD module, designed for deeper layers, follows a similar architecture with some key modifications. It doubles the number of channels while halving the spatial dimensions, which increases the network’s ability to represent more complex features. The DRFD also incorporates GELU activation functions to enhance nonlinear transformations, which are critical for capturing high-level semantic patterns in agricultural scenes.

The core innovation of HRFN is its multi-path fusion strategy. Unlike traditional methods that rely on single convolution operations for downsampling, our approach integrates three complementary mechanisms: CutD preserves spatial structure, ConvD extracts contextual features, and MaxD captures salient patterns. This design ensures that critical information about small weeds is preserved, even as spatial resolution decreases. The hierarchical structure, with SRFD handling low-level details and DRFD processing deeper semantic features, creates a robust feature pyramid that excels at detecting weeds of varying sizes and appearances in complex agricultural environments.

Our experiments show that this multi-path downsampling approach significantly improves small object detection accuracy compared to conventional methods, particularly in challenging scenarios with occlusion, varying lighting, and dense crop backgrounds. The HRFN architecture effectively balances computational efficiency and feature preservation, making it well-suited for practical agricultural applications.

#### 2.4.2 PC-MSFA module

**Figure 5:**
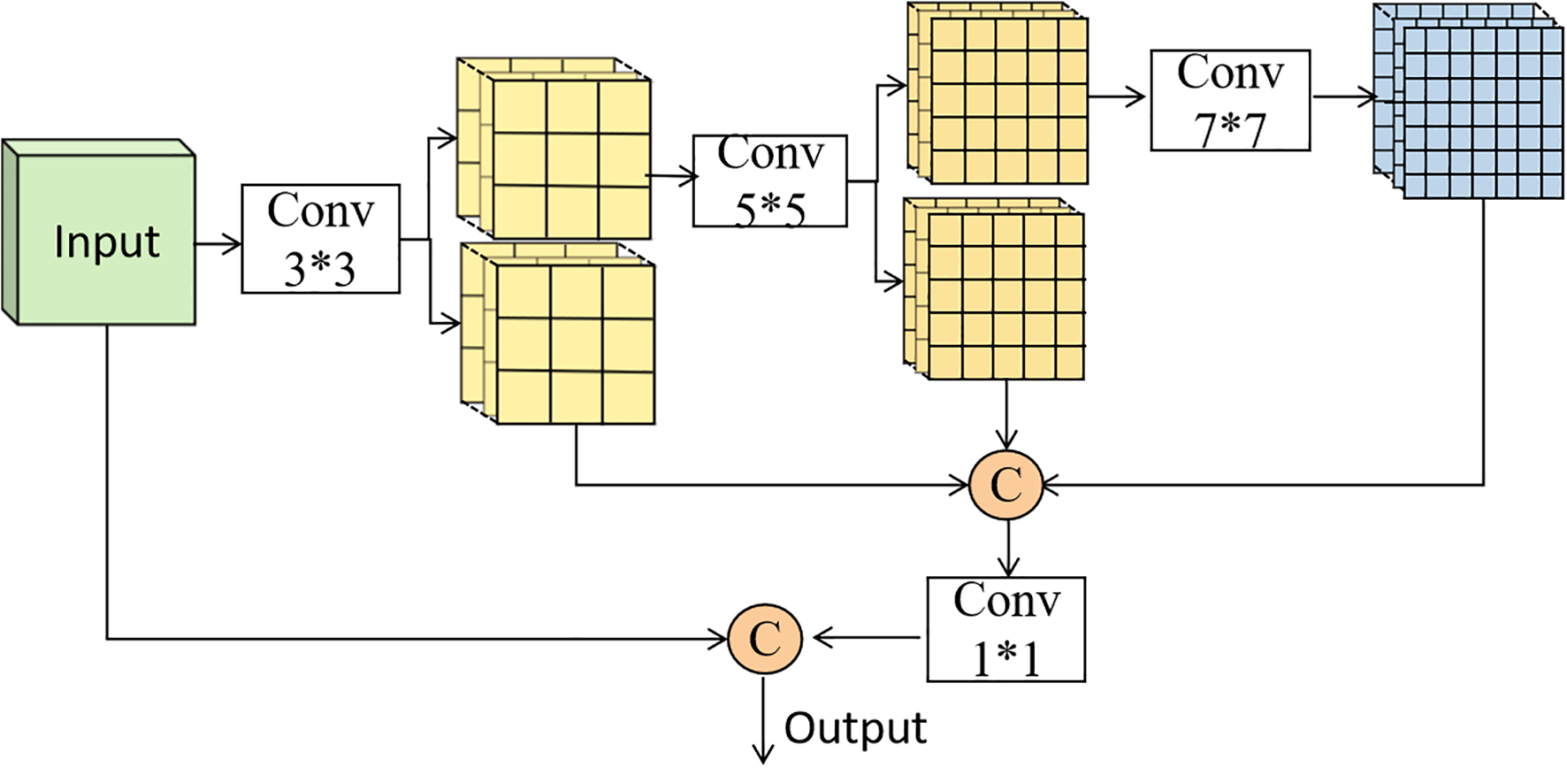
PC-MSFA modules.

The standard C3K2 module in YOLO11 uses fixed-scale convolutions, limiting its ability to detect weeds at different growth stages. To overcome this, we propose the Partial Convolution Multi-Scale Feature Aggregation (PC-MSFA) module, which enhances multi-scale feature extraction efficiently. As shown in **Figure 5**, PC-MSFA employs a hierarchical processing strategy. Initially, the input features undergo a 3×3 convolution for basic feature extraction. These feature maps are then split into subsets: one subset is processed by a 5×5 convolution for medium-scale features, and another by a 7×7 convolution for capturing large-scale context. This partial convolution approach—applying different kernels to specific channel subsets rather than all channels—reduces computational overhead compared to full-scale processing at multiple levels.

The multi-scale features are fused through a 1×1 convolution, then combined with the original features via a residual connection. This design preserves fine-grained details while capturing broader contextual information, essential for detecting weeds at various growth stages, from small seedlings to mature plants.

The key advantage of the PC-MSFA module lies in its efficient multi-scale processing. By selectively applying convolutions to channel subsets, it ensures comprehensive feature coverage without redundant computations. This approach allows the model to capture the morphological variations of weeds across different growth stages while maintaining computational efficiency, making it suitable for real-time field applications.

#### 2.4.3 IntergraDet detection headers

**Figure 6:**
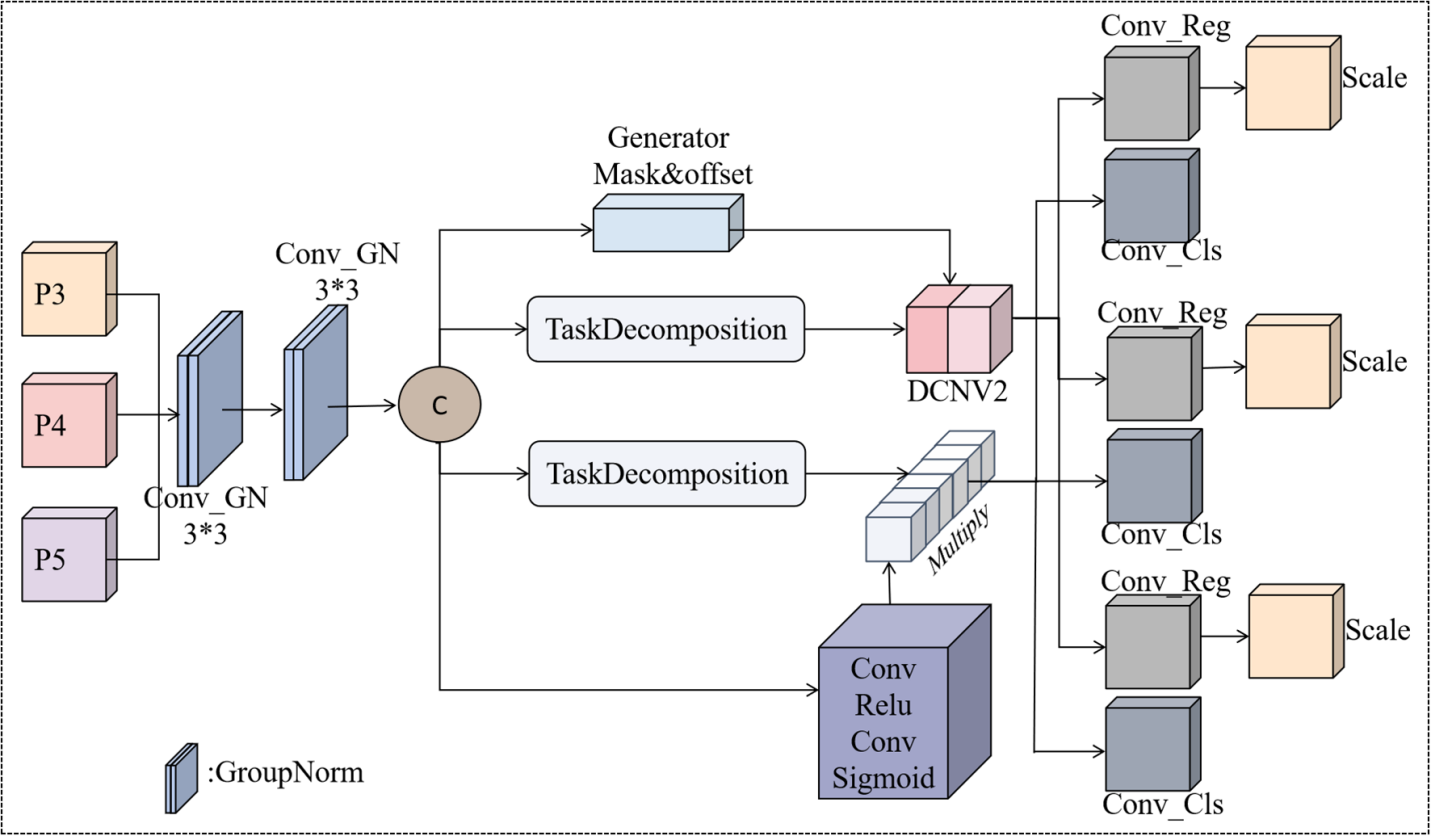
IntergraDet detection headers.


Traditional yolo detection heads process classification and regression tasks independently, leading to task misalignment that reduces performance, especially for small and morphologically similar weeds. To address this, we propose IntegraDet, a task-aligned dynamic detection head that facilitates bidirectional information flow between tasks through a unified processing pipeline. As shown in **Figure 6**, IntegraDet processes multi-scale features from the backbone layers via shared convolutions with GroupNorm activation. These shared features are then decomposed into task-specific representations using an attention-based mechanism (Equation 1):

(1) $$\begin{array}{c} A_{task}=\sigma \left(W_{1}\cdot \delta \left(GAP\left(F\right)\right)\right) \end{array}$$

Here, A_task_ represents the channel attention weights, enabling selective emphasis on task-relevant features. For the regression branch, IntegraDet employs Deformable Convolution v2 to adaptively adjust receptive fields (Equation 2):


(2) $$\begin{array}{c} F_{reg}=DCNv2\left(F,\Delta p,m\right) \end{array}$$

Where Δp and m are dynamically predicted offsets and modulation masks, respectively, allowing precise localization of irregularly shaped weeds. The classification branch uses spatial attention for foreground–background discrimination (Equations 3, 4):


(3) $$\begin{array}{c} F_{cls}=A_{cls}⊙F \end{array}$$

(4) $$\begin{array}{c} A_{cls}=\sigma \left({\text{Conv}}_{1\times 1}\left(F\right)\right) \end{array}$$

Where Acls generates attention maps to suppress background noise. Finally, the predictions are integrated across scales using learnable parameters (Equations 5, 6):


(5) $$\begin{array}{c} Y_{reg}=\sum_{i}\alpha_{i}\cdot {\text{Head}}_{reg}\left(F_{reg,i}\right) \end{array}$$

(6) $$\begin{array}{c} Y_{cls}=\sum_{i}\alpha_{i}\cdot {\text{Head}}_{cls}\left(F_{cls,i}\right) \end{array}$$

where α_i_ adaptively weights contributions from different feature levels.

This design transforms detection by establishing strong connections between classification and localization, eliminating the traditional disconnect between classification confidence and localization precision. The combination of task decomposition, deformable convolutions, and spatial attention mechanisms enhances detection accuracy, particularly for small, densely clustered weeds. It is especially effective in complex agricultural environments where conventional methods struggle with morphologically similar species and varying growth stages.

### 2.5 Evaluation indicators

To comprehensively evaluate the detection performance of the proposed model, we introduce the standard evaluation metrics in object detection (Equations 7–10). The evaluation metrics used in this study include: precision, recall, average precision (AP), and mean average precision (mAP). Precision measures prediction accuracy by calculating the proportion of actual positive samples among all samples predicted as positive. This metric reflects prediction reliability. Recall assesses detection completeness by measuring the proportion of actual positive samples correctly identified. This metric reflects the model’s ability to detect targets. Average precision (AP) comprehensively reflects the model’s detection performance for a single category by calculating the area under the precision-recall curve at various confidence thresholds. Mean Average Precision (mAP) calculates the average of AP values across all categories, providing an overall performance assessment of the model in multi-category detection tasks. This study employs two evaluation criteria: mAP@50 (IoU threshold = 0.5) and mAP@50-95 (IoU thresholds from 0.5 to 0.95 at 0.05 intervals), with the latter providing a stricter performance assessment.


(7) $$\begin{array}{c} \text{Precision}=\frac{\text{TP}}{\text{TP}+\text{FP}} \end{array}$$

(8) $$\begin{array}{c} \text{Recall}=\frac{\text{TP}}{\text{TP}+\text{FN}} \end{array}$$

(9) $$\begin{array}{c} \text{AP}=\int_{0}^{1}\text{p}\left(\text{r}\right)\text{dr} \end{array}$$

(10) $$\begin{array}{c} \text{mAP}=\frac{\text{AP}}{{\text{classes}}_{num}} \end{array}$$

Where TP (True Positive) TP (True Positive) refers to the number of correctly detected targets, i.e., the number of samples predicted by the model to be positive and being positive; FP (False Positive) refers to the number of false positives, i.e., the number of samples predicted by the model to be positive but being negative; FN (False Negative) refers to the number of false negatives, i.e., the number of samples that are positive but not detected by the model.

## 3 Results

### 3.1 Weed detection results based on MMDetection

Weed detection usually starts with the widely used MMDetection framework. Its highly modular code structure allows flexible component combination and replacement, enabling a unified configuration file system. This approach enhances clarity and facilitates extension to other models. We employ two established object detection algorithms, RetinaNet and Faster R-CNN, each integrating a Feature Pyramid Network (FPN) model head with a Resnet50 and Resnet101 backbone. The training process consists of two main phases. We converted LabelMe annotation files to the standard COCO dataset JSON format. We tune hyperparameters, such as learning rate and maximum iterations, to optimize performance and reduce overfitting. **Table 3** shows that both RetinaNet and Faster R-CNN models, using the MMDetection framework, exhibit good performance for weed detection. As the number of iterations increases and with larger backbone models (such as ResNet101), the AP value continues to improve.

**Table 3:** Different models based on MMDetection, training iterations, and model evaluation metrics under the backbone network. In the MMDetection framework, AP stands for Average Precision (mAP).

Model	Backbone	Head	Iterations(steps)	AP (%)	AP50 (%)	AP75 (%)	AP s(%)	APm(%)	APl (%)
RetinaNet	Resnet50	FPN	10000	39.3	55.2	42.0	13.1	35.9	63.9
		FPN	60000	42.2	60.8	45.3	15.0	41.2	68.3
	Resnet101	FPN	10000	39.7	56.0	42.2	12.7	37.4	62.3
		FPN	60000	41.3	60.0	43.3	14.8	39.8	65.9
FasterR-CNN	Resnet50	FPN	10000	42.5	61.1	46.1	17.8	41.4	63.6
		FPN	60000	40.0	60.0	42.7	17.0	39.6	62.6
	Resnet101	FPN	10000	42.8	63.0	45.6	18.8	41.5	61.7
		FPN	60000	39.6	59.3	41.9	15.5	38.7	65.3

### 3.2 The weed detection results based on HDMS-YOLO

The HDMS-YOLO model demonstrates significant performance improvements over the YOLO11n base model, especially in challenging weed categories. As shown in **Table 4**, key performance improvements include increased accuracy and recall for several crops. For Maize, accuracy improved from 90.1% to 91.4%, while recall remained consistent at 91.4%. Similarly, Sugar beet saw its accuracy rise from 86.2% to 88.5%, and recall increased from 88.7% to 89.2%. In Sunflower, accuracy increased from 71.8% to 78.0%, with recall rising from 72.4% to 78.6%. These improvements highlight HDMS-YOLO’s enhanced capability in handling diverse crop categories, even under complex conditions.

**Table 4:** Comparison of weed detection results between YOLO11n and HDMS-YOLO.

		YOLO11n				HDMS-YOLO			
Category	Instances	Precision (%)	Recall (%)	mAP@50 (%)	mAP@50-95 (%)	Precision (%)	Recall(%)	mAP@50 (%)	mAP@50-95 (%)
Maize	1157	90.1	90.6	95.1	73.2	91.4	91.4	95.5	74.3
Sugar beet	1225	86.2	88.7	92.6	76.7	88.5	89.2	92.8	77.4
Sunflower	889	71.8	72.4	75.2	45.3	78.0	78.6	80.8	49.5
Bean	399	88.3	87.2	92.5	79.4	85.3	89.7	92.7	79.8
Pea	55	91.7	89.1	95.1	82.9	94.1	92.7	96.0	82.7
Soy	100	89.9	88.1	90.2	58.1	89.0	89.4	89.4	61.0
Potato	184	87.3	87.5	93.3	76.1	92.1	88.5	94.4	77.8
Pumpkin	120	90.3	93.0	96.4	88.0	91.0	92.2	95.5	89.7
Grasses	4046	68.8	55.6	60.7	28.0	70.2	55.5	62.8	29.6
Thistle	447	59.4	48.4	50.8	30.2	60.6	51.0	53.1	32.2
Geranium	782	64.9	51.7	57.5	38.1	70.6	53.7	62.4	41.7
Knotweed	674	66.8	55.0	62.1	36.8	71.5	58.5	65.5	39.0
Amaranth	140	74.2	82.9	83.9	50.8	79.2	82.9	86.0	51.1
Goosefoot	48.0	29.0	18.8	19.3	13.0	25.4	12.5	15.0	9.7
Potato weed	232	60.8	50.4	54.9	40.8	63.6	52.2	56.5	40.9
Chamomile	485	60.3	48.2	52.2	30.2	58.6	49.7	53.0	30.5
Crucifer	540	74.6	68.7	71.3	51.9	75.7	68.5	73.0	53.4
Plantago	1921	75.3	71.9	77.1	53.9	75.1	73.9	79.6	56.6
Poppy	246	69.4	20.3	36.1	21.2	68.0	31.9	44.8	24.8
Corn spurry	1160	58.4	48.4	49.2	23.2	61.5	47.8	52.4	24.8
Mercuries	449	56.5	53.2	56.9	35.8	70.5	57.5	65.6	41.5
Solanales	257	73.5	72.3	76.4	50.9	82.6	74.0	80.5	54.1
Chickweed	251	68.4	45.0	52.5	27.8	75.6	48.2	59.0	29.2
Labiate	91	63.1	52.6	54.6	24.3	63.7	61.5	62.9	29.7

**Figure 7:**
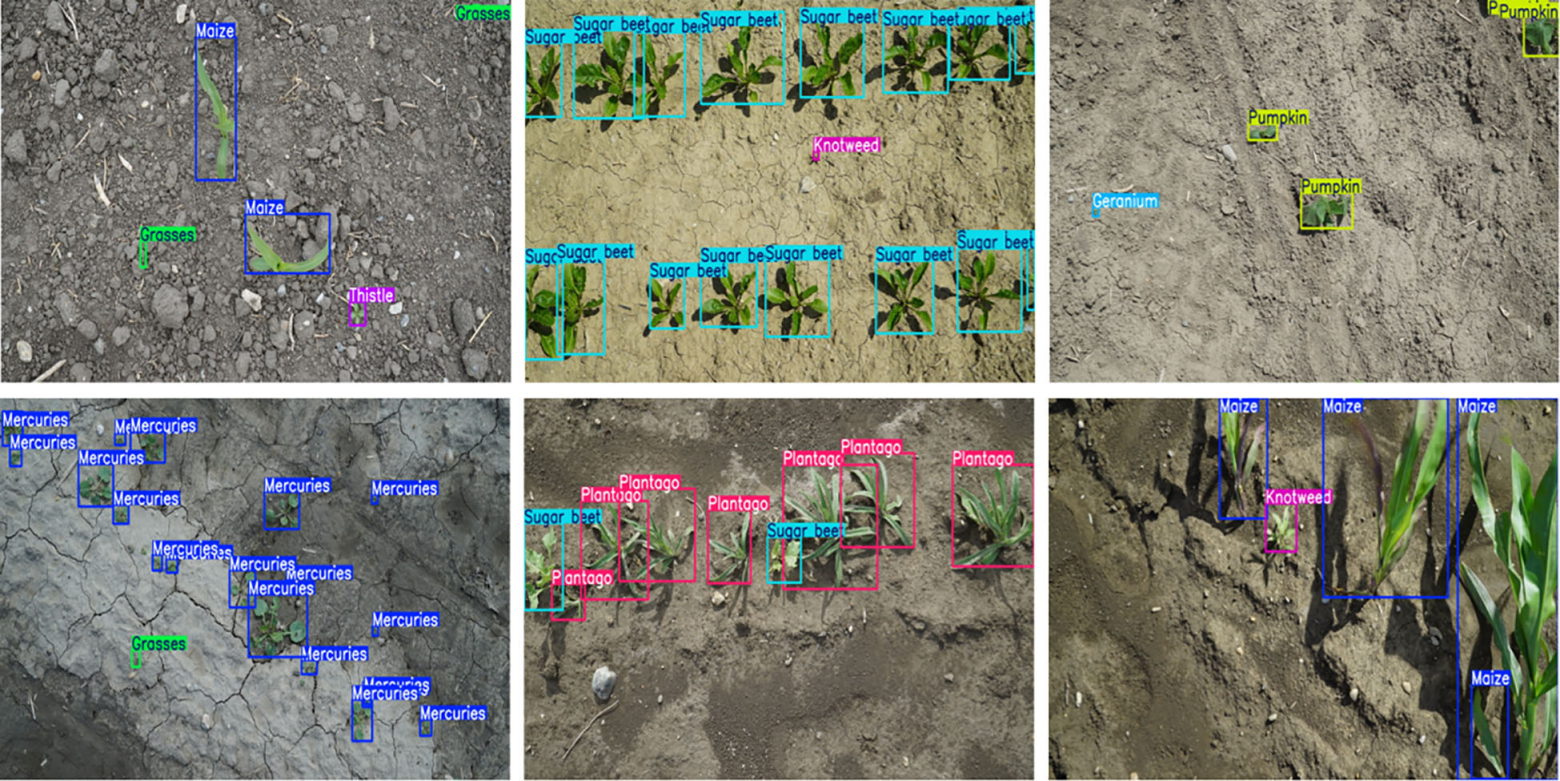
Based on the weed detection results of HDMS-YOLO, the colors of different boxes represent different types of weeds.

In more challenging categories, the model showed notable improvements. Grasses exhibited a performance boost, with accuracy increasing from 68.8% to 70.2%. Geranium saw a significant rise in accuracy, increasing by 5.7 percentage points, from 64.9% to 70.6%, with recall improving by 2.7 percentage points. As shown in **Figure 7**, HDMS-YOLO successfully identified and differentiated various crops and weeds in a real-world agricultural setting, emphasizing its practical application for precision farming.

The model also demonstrated improved performance in small-sample categories. For example, Pea accuracy increased from 91.7% to 94.1%, and Labiate saw mAP50 improve from 54.6% to 62.9%. However, Goosefoot and Poppy showed slight reductions in performance due to limited sample sizes and high visual similarity between species. Despite these minor reductions, HDMS-YOLO exhibited robust performance overall.

The integration of the HRFN and PC-MSFA modules contributed significantly to these results. These modules enhanced precision and recall across both complex and small-sample weed categories, further boosting the model’s adaptability to real-world agricultural environments. For a detailed visualization of the detection results, refer to **Figure 7**, which showcases how HDMS-YOLO distinguishes between different types of weeds and crops in various scenarios.

### 3.3 Ablation experiment results

As summarized in **Table 5**, the ablation experiments demonstrate that each proposed module contributes to performance improvements. HRFN enhances recall, PC-MSFA increases precision and mAP@50, and IntergraDet strengthens both localization and classification. When integrated, these modules provide consistent gains, with the complete HDMS-YOLO model achieving 74.2 percent precision, 66.3 percent recall, an mAP@50 of 71.2 percent, and an mAP@50–95 of 49.2 percent, substantially outperforming the baseline.

**Table 5:** Results of the ablation experiment.

Number	HRFN	PC-MSFA	IntergraDet	Precision(%)	Recal(%)	mAP@ 50(%)	mAP@50-95(%)	FLOPs(G)	FPS(frames/s)	Parameters(×10^6^)
1				71.6	64.2	68.6	47.4	6.3	540	2586832
2	✓			71.6	65.3	69.4	47.7	7.6	436	2594288
3		✓		72.7	64.5	69.6	47.6	7.7	552	2633688
4			✓	72.4	65.0	68.9	47.7	7.9	301	2201699
5	✓	✓		73.0	65.0	70.1	48.0	8.9	443	2641144
6	✓		✓	74.4	65.6	70.1	48.3	9.2	260	2209155
7		✓	✓	72.8	65.4	70.3	48.4	9.3	537	2516072
8	✓	✓	✓	74.2	66.3	71.2	49.2	10.5	263	2270699

In terms of efficiency, the YOLO11 baseline required 6.3G FLOPs and delivered 540 frames per second. The final model increased the computational load to 10.5G FLOPs but remained compact, with 2.27 million parameters corresponding to a size of 4.6 MB, and sustained real-time inference at 263 frames per second, or 6.7 milliseconds per image. These results confirm that the proposed architecture enhances detection accuracy while maintaining efficiency.

**Figure 8:**
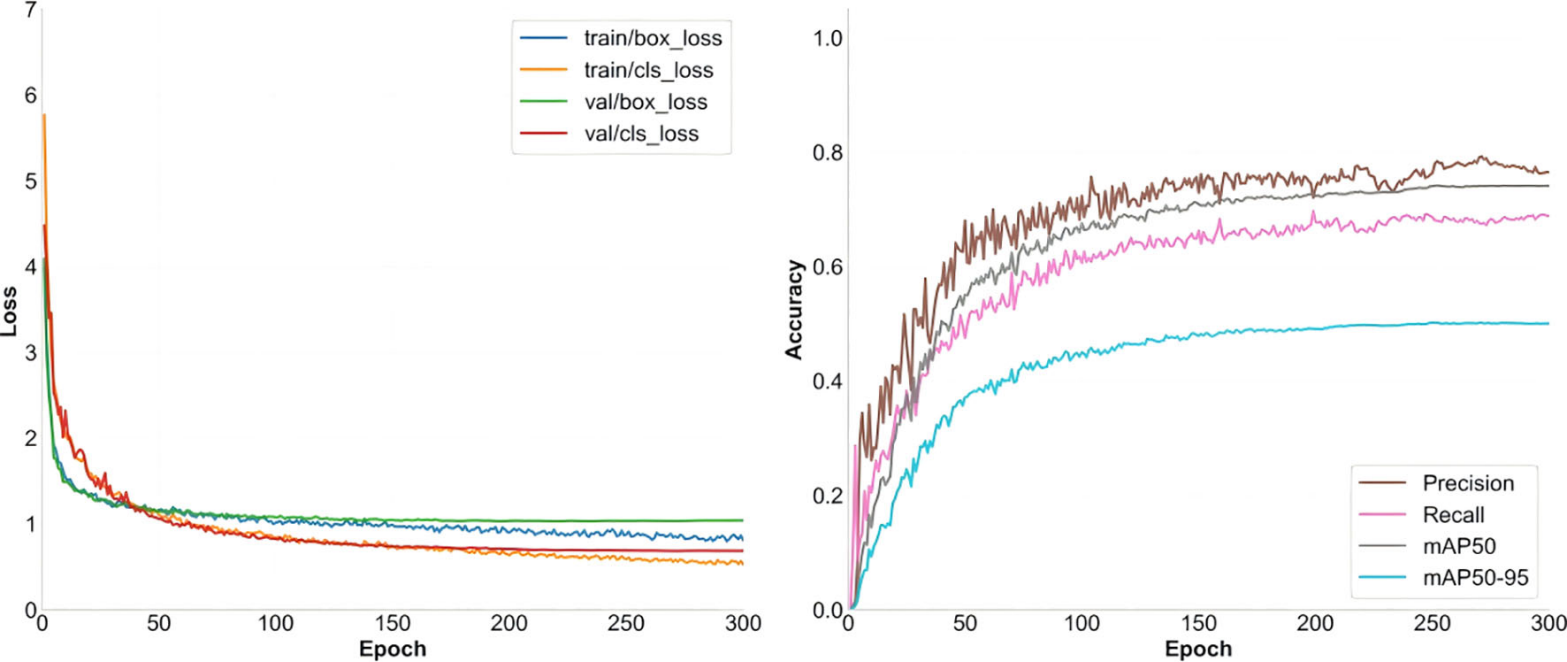
HDMS-YOLO convergence diagram.

The convergence behavior illustrated in **Figure 8** further validates the robustness of the model. Training and validation losses decrease smoothly and align closely in the later stages, while accuracy curves remain stable with minimal fluctuations, demonstrating strong generalization capability.

### 3.4 Model performance comparison results

#### 3.4.1 Comparison results of different models

To comprehensively evaluate the effectiveness of HDMS-YOLO, we compared it with mainstream detectors, optimized variants, and transformer-based models. As shown in **Table 6**, HDMS-YOLO achieved an mAP@50 of 71.2 percent and an mAP@50–95 of 49.2 percent, outperforming lightweight models such as YOLOv8n, YOLOv10n, and YOLOv12n (Talaat and ZainEldin, 2023; Tian et al., 2025; Wang et al., 2024), and maintaining a 0.2-point lead over YOLOv9t (Yaseen, 2024). It also surpassed optimized variants, exceeding the YOLO-CWD model proposed by Ma et al. with 66.9 percent and 45.6 percent, and the YOLO-CBAM model proposed by Wang et al. with 70.5 percent and 48.4 percent (Ma et al., 2025; Wang et al., 2022). Compared with transformer-based detectors, HDMS-YOLO approached the accuracy of DINO (Zhang et al., 2022), which reached 50.2 percent mAP@50-95, and outperformed Deformable DETR (Zhu et al., 2020) with 47.6 percent, while requiring only 10.5 GFLOPs, far less than their 178.5 and 173 GFLOPs.

**Table 6:** Performance comparison results of different models in ablation experiments.

Model	Backbone	Image size (px)	mAP50 (%)	mAP50-95 (%)	Parameters (M)	FLOPS (G)
HDMS-YOLO	**Ours**	**640×640**	**71.2**	**49.2**	2.27	10.5
YOLOv8n	C2f CSPDarkNet	640×640	66.5	45.2	3.01	8.7
YOLOv9t	PGI and GELAN	640×640	68.0	49.0	**2.0**	7.7
YOLOv10n	Improved CSPNet	640×640	67.1	47.2	2.27	6.7
YOLO11n	C3k2 CSPDarkNet	640×640	68.6	47.4	2.59	**6.3**
YOLOv12n	R-ELAN	640×640	67.2	46.3	2.56	6.5
RetinaNet	Resnet50	640×640	54.7	39.6	36.8	81.69
RetinaNet	Resnet101	640×640	55.2	40.2	55.8	85.41
YOLO-CWD*[1]	C2f ECSADarkNet	640×640	66.9	45.6	3.5	9.6
YOLO-CBAM*[50]	Bottleneck_CSP	640×640	70.5	48.4	53.3	135.1
Deformable DETR*[25]	ResNet-101	640×640	65.8	47.6	40.1	173
DINO*[29]	ResNet-50	640×640	**71.2**	**50.2**	47.5	178.5
Faster R-CNN	Resnet50	640×640	62.9	45.1	41.5	78.12
Faster R-CNN	Resnet101	640×640	64.4	46.2	60.5	81.77

In the table, the best values for the HDMS-YOLO model have been bolded; however, the optimal values for Parameters (M) and FLOPS (G) correspond to YOLOv9t and YOLO11n, respectively.

**Figure 9:**
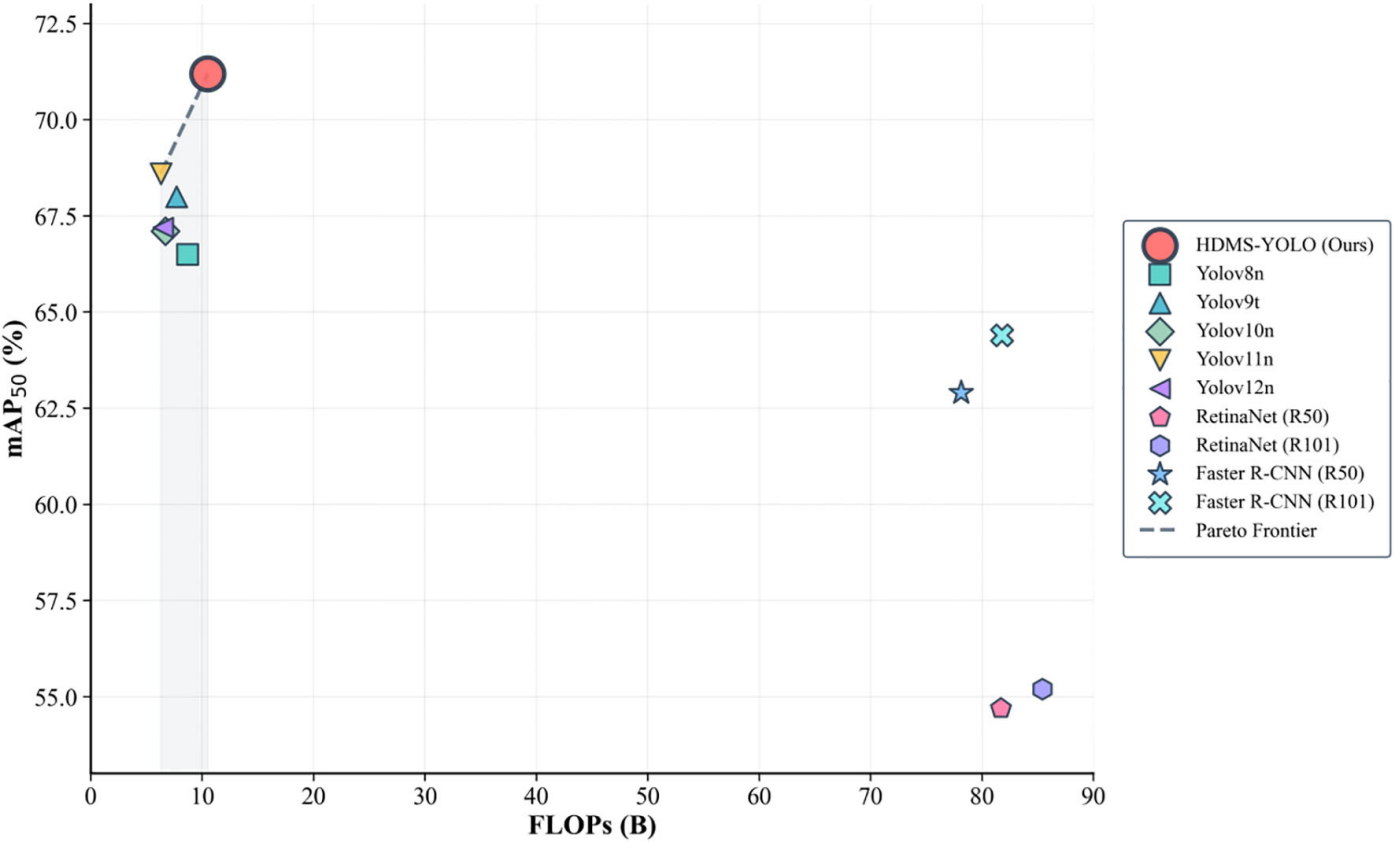
Comparison of performance and FLOPs among different models.

Beyond accuracy, HDMS-YOLO also demonstrates a favorable trade-off between precision and complexity. As shown in **Figure 9**, it achieved the highest mAP@50 among lightweight YOLO models while maintaining low FLOPs, improving by about 3 percent over YOLOv11n without a noticeable increase in computation. Unlike two-stage detectors such as RetinaNet and Faster R-CNN, which demand much higher computational cost but deliver lower accuracy, HDMS-YOLO thus proves particularly effective in low-resource environments. Its efficiency is further supported by the results in **Table 7**, where the model completed evaluation in 18 seconds with an inference time of 6.8 milliseconds per image. Although YOLOv11n achieved a slightly shorter total time of 17 seconds, its longer postprocessing offset the gain, and the marginal inference difference is negligible given the superior accuracy of HDMS-YOLO.

**Table 7:** Speed indicators and time Predictions of YOLO11n and HDMS-YOLO.

Model	Preprocess (ms)	Inference (ms)	Postprocess (ms)	Test time (s)
Yolo 11n	0.2	2.8	**1.7**	17
HDMS-YOLO	**0.2**	**6.8**	0.9	**18**

The best values in each column of the table have been highlighted in bold.

**Figure 10:**
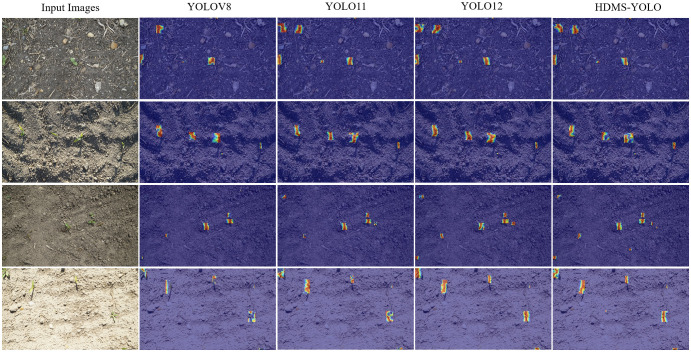
Comparison of the weed detection effects of different models.

**Figure 10** visually compares the weed detection effects of HDMS-YOLO with other models (YOLOv8, YOLO11, YOLO12). The results demonstrate that HDMS-YOLO outperforms multiple detection models, exhibiting a low missed detection rate and enhanced capability to detect small-target weeds. Nevertheless, accurate weed-crop detection remains challenging due to the insufficient availability of mixed samples of specific weed species with crops and the inherent difficulty in identifying small-target weeds.

#### 3.4.2 Model performance analysis

**Figure 11:**
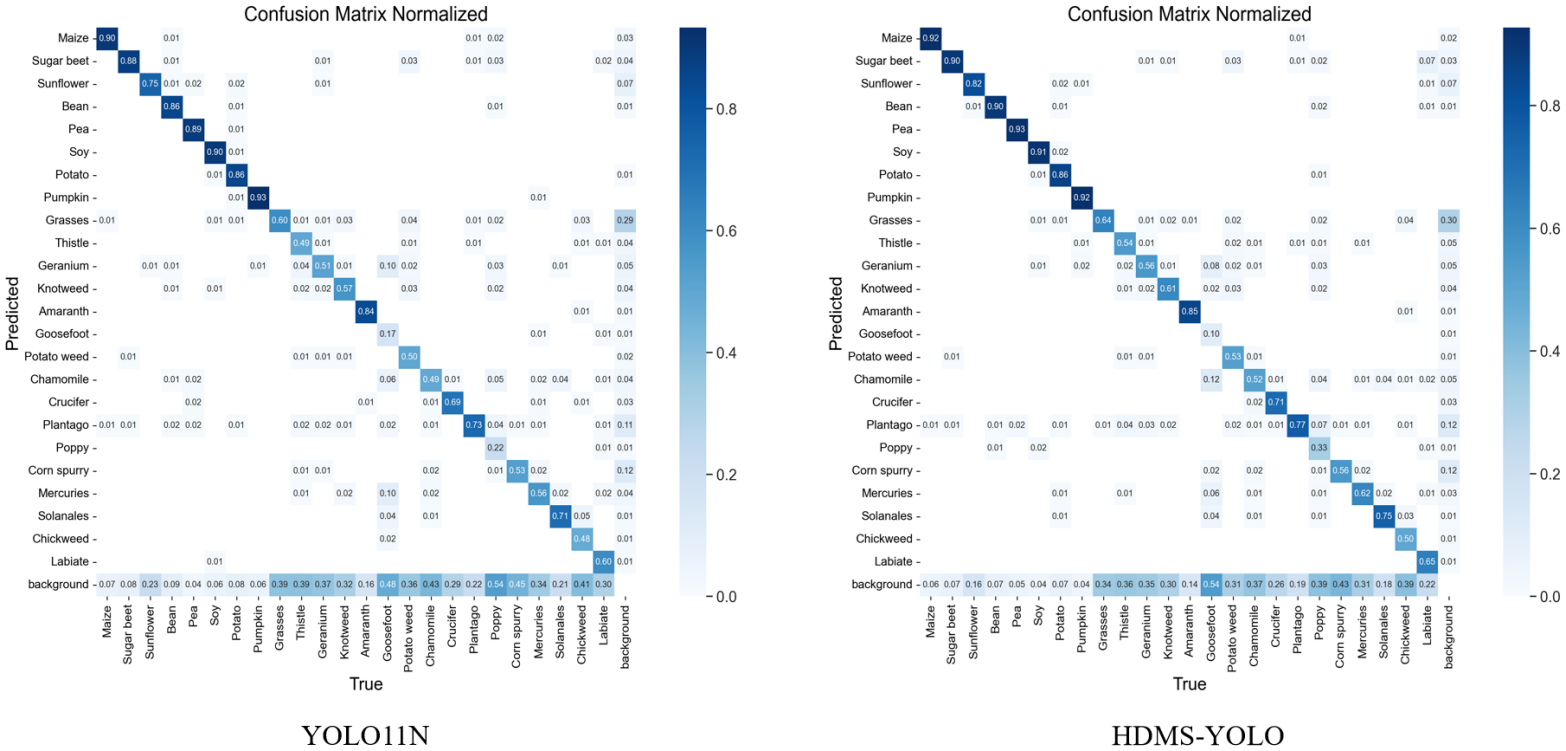
Confusion matrix of the model’s classification results on the dataset. Here, **(a)** is the confusion matrix diagram of YOLO11N, and **(b)** is the confusion matrix diagram of HDMS-YOLO.

As shown in **Figure 11**, the confusion matrices provide a comparative analysis of the classification results between the YOLO11 base model and HDMS-YOLO on the Weed dataset. The YOLO11 model performs well in most categories, but notable misclassifications are observed. For example, Grasses and Sunflower are frequently confused with Soy, as indicated by the recall values of 55.6% and 72.4%, respectively. The background class also shows significant overlap with Grasses and Thistle, leading to missed detections. This suggests that the model struggles with distinguishing between weeds and crops with similar visual features, particularly in complex or cluttered scenes.

In contrast, the HDMS-YOLO model shows clear improvements. The confusion matrix for HDMS-YOLO reveals higher precision and recall across multiple categories. For example, Grasses and Sunflower show increased precision, with Grasses reaching 60% and Sunflower improving to 78%, compared to 55% and 50% for the YOLO11 model. Additionally, Thistle and Geranium benefit from improved recall, with Thistle rising from 48.4% to 51.0% and Geranium from 51.7% to 53.7%, highlighting the model’s enhanced ability to handle these more challenging categories.

**Figure 12:**
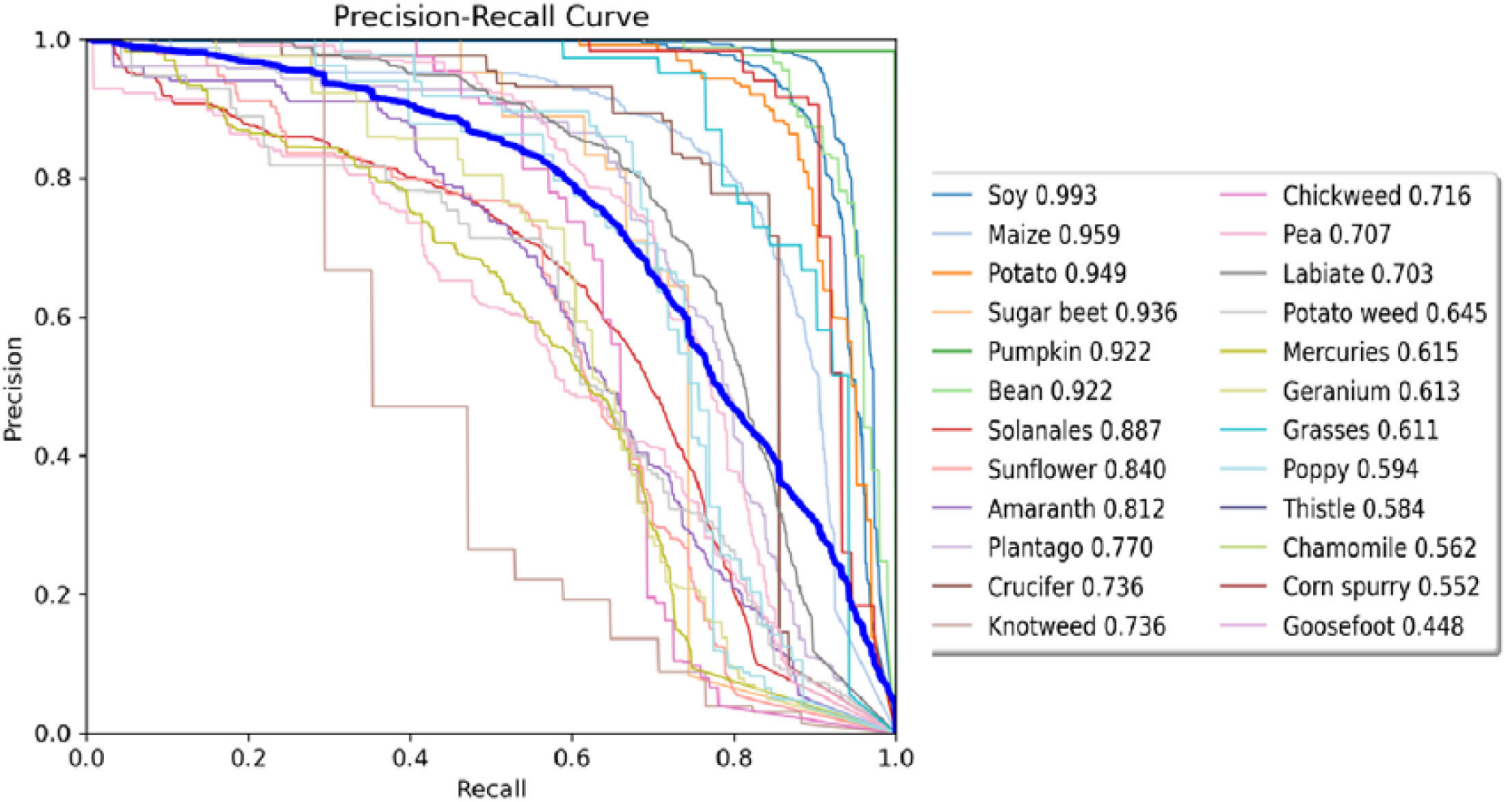
Precision-recall curve chart.

The precision-recall curve in **Figure 12** further illustrates HDMS-YOLO’s improved performance. Soy stands out with 99.3% precision and recall close to 1.0, demonstrating near-perfect detection. Maize and Potato also show strong performance, with precision values of 95.9% and 94.9%, respectively. However, classes like Grasses (precision = 61.1%, recall = 39%) and Chickweed (precision = 71.6%, recall = 52%) show lower performance, primarily due to their visual similarity to other species and limited sample sizes in the dataset. The Poppy and Goosefoot classes exhibit even lower recall, with Poppy at 31% and Goosefoot at 25%, which reflects challenges related to class imbalance and visual similarity to other weeds.

These quantitative results confirm that HDMS-YOLO significantly improves over YOLO11, particularly in terms of precision and recall across various crop and weed categories. However, further improvements could be made in handling underrepresented classes. Techniques such as data augmentation or few-shot learning could address the performance issues with Goosefoot, Poppy, and Grasses, enhancing the model’s robustness across all categories.

## 4 Discussion

Weed detection is crucial in agriculture, as it helps prevent a reduction in crop yields, which leads to an estimated global loss of $32 billion annually (Kubiak et al., 2022). Traditional weed control methods, including manual labor and chemical applications, are labor-intensive, expensive, and environmentally damaging. Automated systems, like the HDMS-YOLO model, provide a more sustainable and efficient solution, capable of detecting weeds in real-time and guiding precision herbicide application. While HDMS-YOLO performs well on the CropAndWeed dataset, several challenges remain, particularly in detecting small, crowded, or similar-looking weeds.

The current model significantly improves performance over earlier versions, such as YOLOv8n and YOLOv10n, with HDMS-YOLO achieving 71.2% mAP@50 and 49.2% mAP@50-95. However, as observed, small and similar-looking weeds, such as Goosefoot and Poppy, showed low recall rates (15.0% and 44.8%, respectively). This issue is largely due to class imbalance, where underrepresented species, particularly those with limited training data, struggle to achieve high accuracy. These findings are consistent with previous research by Veeragandham et al, where models like VGG-19 achieved high accuracy on larger datasets but struggled with rare classes, particularly when there is class imbalance and limited data for certain species (Veeragandham and Santhi, 2022). The limited data for rare weeds leads to overfitting, a challenge that affects models trained on skewed datasets.

In comparison to two-stage models like Faster R-CNN, HDMS-YOLO shows a computational advantage, requiring only 10.5 GFLOPs as opposed to the 78.12 GFLOPs required by Faster R-CNN. However, HDMS-YOLO still faces challenges in high-density weed environments, where weeds of similar size and shape are often clustered together. Transformer-based models, such as Deformable DETR, offer stronger performance in such situations, but their computational demands may make them unsuitable for real-time applications. Few-shot learning and domain adaptation, as explored by Li and Fan et al, could be employed to address the problem of underrepresented classes in our model by augmenting the dataset with synthetic data or by transferring knowledge from similar tasks (Li et al., 2024; Fan et al., 2024).

The limitations of HDMS-YOLO primarily lie in its generalizability to different environments and handling rare classes. Although the model performs well in the CropAndWeed dataset, its performance may degrade in real-world settings, where lighting conditions, weed species, and crop types vary significantly. This issue of generalizability is a common problem in AI models, as demonstrated in studies like Xu et al, which proposed W-YOLOv5 for crop detection, emphasizing the challenge of adapting models to new environments (Xu et al., 2024). Furthermore, the risk of overfitting remains a concern, particularly for models trained on datasets with imbalanced class distributions.

For future work, we propose integrating multi-sensor fusion, combining visual, thermal, and LiDAR data to improve detection under poor visibility or occluded conditions. Temporal tracking of weed growth, as explored by Goyal et al, could also enhance the model’s ability to monitor weeds over time and differentiate between weeds at various growth stages (Goyal et al., 2025). Additionally, field deployment strategies, such as real-time decision-making for robotic systems, are essential for applying HDMS-YOLO in agricultural practices. Implementing few-shot learning techniques and incorporating domain adaptation strategies will further improve the model’s generalizability to new environments and rare species.

## 5 Conclusion

In this study, we proposed HDMS-YOLO, an improved detection model based on the YOLOv11 architecture, to address the complex challenge of recognizing multiple weed and crop types in farmland. A hierarchical feature processing mechanism was constructed by introducing the shallow feature structure reconstruction module (SRFD) and the deep feature dynamic reconstruction module (DRFD), significantly enhancing multi-scale object perception. The PC-MSFA module significantly enhanced the detection performance of weeds across various scales through cross-stage partial connection and progressive multi-scale feature aggregation. Furthermore, the dynamic task alignment detection head (IntegraDet) adaptively adjusted classification and regression task weights, improving discrimination between morphologically similar crops and weeds. Experimental results demonstrated that HDMS-YELO achieved notable performance on the CropAndWeed dataset, with 74.2% precision, 66.3% recall, 71.2% mAP@50, and 49.2% mAP@50-95, requiring only 2.27 million parameters. The combination of high accuracy and low parameter complexity provides practical technical support for deployment on embedded devices and intelligent weeding robot systems, significantly advancing agricultural automation.

## Data Availability

The datasets presented in this study can be found in online repositories. The names of the repository/repositories and accession number(s) can be found in the article/supplementary material.
